# Evaluation of Tumor-Targeting Properties of an Antagonistic Bombesin Analogue RM26 Conjugated with a Non-Residualizing Radioiodine Label Comparison with a Radiometal-Labelled Counterpart

**DOI:** 10.3390/pharmaceutics11080380

**Published:** 2019-08-02

**Authors:** Maryam Oroujeni, Ayman Abouzayed, Fanny Lundmark, Bogdan Mitran, Anna Orlova, Vladimir Tolmachev, Ulrika Rosenström

**Affiliations:** 1 Department of Immunology, Genetics and Pathology, Uppsala University, SE-751 85 Uppsala, Sweden; 2 Department of Medicinal Chemistry, Uppsala University, SE-751 23 Uppsala, Sweden; 3 Science for Life Laboratory, Uppsala University, SE-750 03 Uppsala, Sweden

**Keywords:** prostate cancer, bombesin antagonistic analogue, GRPR, RM26, tyrosine, PC-3 xenografts

## Abstract

Radiolabelled antagonistic bombesin analogues are successfully used for targeting of gastrin-releasing peptide receptors (GRPR) that are overexpressed in prostate cancer. Internalization of antagonistic bombesin analogues is slow. We hypothesized that the use of a non-residualizing radioiodine label might not affect the tumour uptake but would reduce the retention in normal organs, where radiopharmaceutical would be internalized. To test this hypothesis, tyrosine was conjugated via diethylene glycol linker to N-terminus of an antagonistic bombesin analogue RM26 to form Tyr-PEG_2_-RM26. [^111^In]In-DOTA-PEG_2_-RM26 was used as a control with a residualizing label. Tyr-PEG_2_-RM26 was labelled with ^125^I with 95% radiochemical purity and retained binding specificity to GRPR. The IC_50_ values for Tyr-PEG_2_-RM26 and DOTA-PEG_2_-RM26 were 1.7 ± 0.3 nM and 3.3 ± 0.5 nM, respectively. The cellular processing of [^125^I]I-Tyr-PEG_2_-RM26 by PC-3 cells showed unusually fast internalization. Biodistribution showed that uptake in pancreas and tumour was GRPR-specific for both radioconjugates. Blood clearance of [^125^I]I-Tyr-PEG_2_-RM26 was appreciably slower and activity accumulation in all organs was significantly higher than for [^111^In]In-DOTA-PEG_2_-RM26. Tumor uptake of [^111^In]In-DOTA-PEG_2_-RM26 was significantly higher than for [^125^I]I-Tyr-PEG_2_-RM26, resulting in higher tumour-to-organ ratio for [^111^In]In-DOTA-PEG_2_-RM26 at studied time points. Incorporation of amino acids with hydrophilic side-chains next to tyrosine might overcome the problems associated with the use of tyrosine as a prosthetic group for radioiodination.

## 1. Introduction

Prostate cancer (PC) is the second leading cause of cancer-related fatality in men [1]. The treatment of disseminated castration-resistant PC requires a systemic targeting approach. As the androgen receptor (AR) is the main oncogenic driver in PC, most drugs for its treatment are used to inhibit AR activity. However, since resistance to both first- and second-line androgen deprivation therapies (ADT) is a common problem, development of drugs towards other targets for visualization and treatment of PC is of utmost importance [2,3].

Targeting of prostate-specific membrane antigen (PSMA) is currently the major focus in development of a targeted radionuclide therapy of PC and its metastases [4,5]. There is no known natural ligand for PSMA, and the reason for PSMA upregulation in PC remains unknown [6,7,8]. The PSMA is highly expressed on prostate epithelial cells and strongly upregulated in PC. However, PSMA is not specific to the prostate gland and is expressed in other normal tissues (salivary glands, duodenal mucosa, and subpopulation of neuroendocrine cells in the colonic crypts) and neoplastic tissues. Xerophthalmia and xerostomia are often observed side-effects of PSMA-targeted radionuclide therapy inhibitors because of accumulation in salivary and lacrimal glands [4,5]. Therefore, it is desirable to develop agents targeting other PC-specific molecular alterations.

A possible therapeutic target in PC is gastrin-releasing peptide receptor (GRPR/BB2), a membrane bound G-protein coupled receptor. This receptor is a member of the bombesin receptor family together with neuromedin B receptor (NMBR/BB1), bombesin receptor subtype 3 (BB3) and subtype 4 (BB4). Overexpression of GRPR has been reported in PC amongst other cancer forms [9]. This, together with the low GRPR expression in healthy tissue, makes it a promising target in PC.

The use of peptide ligands and their analogues for tumour targeting has shown advantages such as excellent vascular permeability and rapid penetration into tumour [10]. Gastrin-releasing peptide (GRP) derivatives have shown high affinity to GRPR and are used in clinical studies [11]. Bombesin (BN) is a linear tetradecapeptide, an analogue of the mammalian GRP, which binds to GRPR with high affinity [12]. Since bombesin was assumed to be more stable than GRP, a number of bombesin-based targeting agents have been extensively investigated in preclinical and clinical studies for imaging and therapy [13,14,15,16,17].

Peptide-based agonists have been considered earlier as more preferable targeting agents compared to antagonists, because the rapid internalization of agonists along with residualizing radiometal labels could provide a high tumour accumulation, resulting in high tumour-to-background ratios [18,19,20]. The major disadvantage is systemic physiologic action. To overcome the disadvantages associated with agonist-based bombesin analogues, antagonists were introduced [21,22]. Antagonistic bombesin-based peptides have shown several other advantages compared to agonists such as more favourable biodistribution and targeting properties, and more binding sites available for targeting than agonists, which may make them preferable over agonists for in vivo tumour targeting [23,24,25,26,27].

Radiometal-labelled antagonistic bombesin analogues have demonstrated efficient targeting of GRPR-expressing tumours. However, internalization of bombesin antagonists by cancer cells was slow. Thus, the use of residualizing properties of a label should not be critical for efficient tumour targeting. We have shown earlier for slowly internalizing scaffold-protein-based agents that the use of non-residualizing labels offers advantage of rapid release of radionuclide from normal tissues after off-target interactions followed by internalization of targeting agents [28,29]. We hypothesized that the residualizing properties of the label for GRPR antagonist are not critical for a good tumour retention but a non-residualizing label would provide quicker clearance from normal tissues.

A possible therapeutic nuclide providing a non-residualizing label is iodine-131, a medium-energy beta-emitter. In preclinical studies, iodine-131 is often substituted by the convenient surrogate, iodine-125, a low energy gamma emitter with a half-life of 59.4 days. Radioiodine can be covalently bound to targeting proteins and peptides containing an activated phenolic ring, for example, a tyrosine residue using an electrophilic reaction [30]. Since the bombesin does not contain any tyrosine, or other moiety with activated phenolic ring, an incorporation of tyrosine is desirable for iodination. 

Several studies have shown that the antagonistic bombesin analogue RM26 (d-Phe-Gln-Trp-Ala-Val-Gly-His-Sta-Leu-NH_2_) [31], labelled with different radionuclides such as ^68^Ga [32,33,34], ^111^In [24,32], ^18^F [35], ^55/57^Co [36], and ^177^Lu [37] containing different chelators, is a potential targeting agent for in vivo targeting of GRPR-expressing tumours. Previous structure-activity studies have demonstrated that modification at C-terminal region of bombesin resulted in dramatic reduction of the receptor binding affinity [38]. Therefore, the N-terminal of these analogues was used for coupling to the chelator for loading with radiometal [22,39]. For conjugation of chelators, a PEG_2_-spacer was introduced at N-terminus of RM26. This linker prevents interference of a nuclide-chelator complex in binding of RM26 to the receptors [40]. For development of radioiodine labelled analogue, we have conjugated a tyrosine instead of chelator to the PEG_2_ linker.

**Figure 1:**
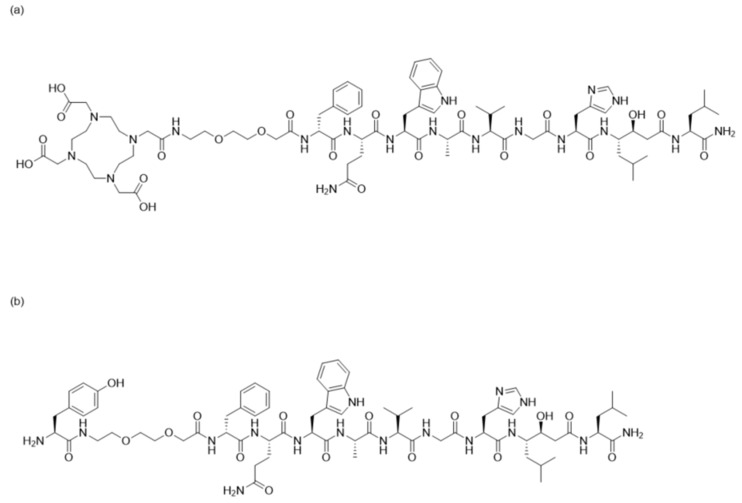
Structural formula of (**a**) DOTA-PEG_2_-[D-Phe^6^, Sta^13^, Leu^14^] bombesin[6–14] (DOTA-PEG_2_-RM26); (**b**) Tyrosine-PEG_2_-[D-Phe^6^, Sta^13^, Leu^14^] bombesin[6–14] (Tyr-PEG_2_-RM26).

The goal of this study was to evaluate the tumour-targeting properties of an antagonistic bombesin analogue labelled with non-residualizing (^125^I-Tyr) and labels residualizing (^111^In-DOTA). For this purpose, the nine-amino-acid bombesin analogue RM26 was synthesized and N-terminus conjugated to tyrosine (Tyr) or DOTA via the diethylene glycol chain (PEG_2_) to form Tyr-PEG_2_-RM26 and DOTA-PEG_2_-RM26, respectively (Figure 1). Tyr-PEG_2_-RM26 was labelled with radiohalogen iodine-125 and in vitro and in vivo properties of [^125^I]I-Tyr-PEG_2_-RM26 were studied and compared to properties of [^111^In]In-DOTA-PEG_2_-RM26 having a residualizing label.

## 2. Materials and Methods 

### 2.1. Peptide Synthesis and Characterization

The bombesin analogue Tyr-PEG_2_-[d-Phe^6^, Sta^13^, Leu^14^] bombesin [6,7,8,9,10,11,12,13,14] (denoted as Tyr-PEG_2_-RM26) was synthesized by manual solid-phase peptide synthesis (SPPS) using standard Fmoc/t-Bu conditions, similar to previously described method [32]. The identity and purity was confirmed by analytical HPLC/ESI-MS using a Dionex UltiMate 3000 HPLC (Thermo Fisher Scientific, Waltham, MA, USA) system with a Bruker amazon SL ion trap mass spectrometer and detection by UV (diode array detector, 214, 254 and 280 nm). The crude Tyr-PEG_2_-RM26 was purified using reversed-phase high-performance liquid chromatography (RP-HPLC) (UV-triggered (220 nm) fraction collection with a Dionex UltiMate 3000 HPLC system, using an Macherey-Nagel Nucleodur C18 column (21 × 125 mm, 5 µm particle size).

DOTA-PEG_2_-[d-Phe^6^, Sta^13^, Leu^14^] bombesin [6,7,8,9,10,11,12,13,14] (denoted as DOTA-PEG_2_-RM26) was synthesized and characterized as previously described [34].

### 2.2. Radiolabelling and In Vitro Stability Test

For labelling with ^125^I, Tyr-PEG_2_-RM26 was reconstituted in Milli-Q water to obtain a concentration of 0.7 mg/mL (stock solution). Then, Tyr-PEG_2_-RM26 solution (2 µL, 1 nmol) was mixed with 40 µL PBS in a Pierce Iodination Tube (Thermo Fisher Scientific, Waltham, MA, USA), which activates iodine-125. 4 µL of ^125^I-sodium iodide (8–15 MBq) was added. The reaction mixture was incubated at room temperature for 15 min. Radiochemical yield was determined using high-performance liquid chromatography (HPLC), conducted on an Elite LaChrom system (Hitachi, VWR, Darmstadt, Germany) consisting of an L-2130 pump, a UV detector (L-2400), and a radiation flow detector (Bioscan, Washington, DC, USA) coupled in series. The radiolabelled conjugate was isolated by preparative-HPLC. HPLC conditions were as follows: A = 10 mM TFA/H_2_O; B = 10 mM TFA/acetonitrile (MeCN); UV-detection at 220 nm; gradient elution: 0–15 min at 5 to 70% B, 15–18 min at 70 to 95% B, 19–20 min at 5% B; and flow rate was 1.0 mL/min. The labelled compound was separated using an analytical column (Phenemenex, Aschaffenburg, Germany; Luna^®^ 5 µm C18, 100 Å; 150 × 4.6 mm column). After isolation, radiolabelled conjugate was purified using Sep-Pak^®^ Vac RC C8 cartridge (Waters, Milford, MA, USA). The cartridge was activated with 10 mL of 90% EtOH/water followed by passing 10 mL Milli-Q water. Then, the radiolabelled mixture was loaded. The cartridge was rinsed by passing 10 mL of Milli-Q water. The radiolabelled conjugate was purified by passing 1 mL of 90% EtOH/water and the first 6–7 drops coming from syringe connected to cartridge was collected. The purity of the labelled conjugate was determined by HPLC.

The labelling of DOTA-PEG_2_-RM26 with ^111^In was performed based on previously developed protocol [41]. Briefly, 92 µL of 0.2 M ammonium acetate buffer pH 5.5 was added to aqueous solution of DOTA-PEG_2_-RM26 (2 µL, 2 nmol) followed by addition of ^111^In-indium chloride solution (30–40 µL, 10~20 MBq). The reaction mixture was incubated at 85 °C for 30 min. The radiochemical yield and purity were analysed using instant thin-layer chromatography (ITLC) strips using citric acid (0.2 M, pH 2.0) as the running solution.

In vitro stability of [^125^I]I-Tyr-PEG_2_-RM26 was evaluated in PBS and in presence of 100-fold molar excess of sodium iodide (1 mM). After purification, samples of freshly labelled conjugate (10 µL, 0.01 nmol) were mixed with 1 µL of NaI and incubated at room temperature for 1 h. Control samples were mixed with equal volume of PBS. The experiment was performed in duplicates. The stability of [^125^I]I-Tyr-PEG_2_-RM26 was analyzed by HPLC.

### 2.3. Lipophilicity Assay: LogP Measurements

Lipophilicity was determined as the logarithm of partition coefficient, log*P*, of the radiolabelled compound between *n*-octanol and water [42]. To determine the lipophilicity of both radioconjugates, 500 µL of *n*-octanol was added to a tube containing the same volume of Milli-Q water. 1 pmol (10 kBq) of radioconjugate was added. The mixture was vigorously vortexed for 1–2 min and then centrifuged. Aliquots of 100 μL were taken from each phase and their radioactivity was measured by an automated gamma-counter. The partition coefficient was calculated as the average log of a ratio of the radioactivity in organic and aqueous fractions. Each measurement was repeated in triplicate.

### 2.4. In Vitro Studies

GRPR-expressing human PC cell line PC-3 and DU-145 cell lines (ATCC) were cultured in RPMI media complemented with 10% FBS, 2 mM l-glutamine, and PEST (penicillin 100 IU/mL) (all from Biochrom AG, Berlin, Germany). This media is referred to as complete media in the text. In all in vitro experiments, cells were incubated in complete media. During in vitro specificity experiments, cells were detached using trypsin-EDTA solution (0.25% trypsin, 0.02% EDTA in buffer; Biochrom AG). All experiments were performed in triplicate and 1 × 10^6^ cells/dish were seeded one day before the experiment.

#### 2.4.1. In Vitro Binding Specificity Assay

The in vitro binding specificity test of [^125^I]I-Tyr-PEG_2_-RM26 was performed on PC-3 and DU-145 cell lines. A set of three dishes containing approximately 10^6^ cells/dish was seeded. The cells were incubated with 1 nM concentration of [^125^I]I-Tyr-PEG_2_-RM26 solution for 1 h at 37 °C. To saturate GRPR, one set of dishes was treated with 200-fold molar excess of non-labelled peptide, NOTA-PEG_4_-RM26, 15 min before adding the radiolabelled compound. After washing with serum-free media, cells were detached by treatment with 1 mL trypsin-EDTA solution twice. Cell-associated radioactivity was measured in the gamma-counter and presented as percentage of cell-associated activity.

#### 2.4.2. In Vitro Competitive Binding Assay: IC_50_ Determination

The in vitro competitive binding assay was performed using [^111^In]In-NOTA-PEG_4_-RM26. The half maximal inhibitory concentration (IC_50_) was determined for non-labelled Tyr-PEG_2_-RM26 and DOTA-PEG_2_-RM26 conjugates. A set of three dishes containing approximately 10^6^ cells/dish was used. Cell monolayers were incubated with increasing concentrations of Tyr-PEG_2_-RM26 and DOTA-PEG_2_-RM26 (0–270 nM) in the presence of [^111^In]In-NOTA-PEG_4_-RM26 (1 nM) for 5 h at 4 °C. After incubation, the cells were treated with trypsin–EDTA solution at 37 °C, collected and cell-associated radioactivity was determined. The IC_50_ values were calculated by fitting the data by nonlinear regression using GraphPad Prism software (GraphPad Software Inc., San Diego, CA, USA).

#### 2.4.3. Cellular Processing Assay

The PC-3 cells (10^6^ cells/dish) were incubated with 1 nM of [^125^I]I-Tyr-PEG_2_-RM26 or [^111^In]In-DOTA-PEG_2_-RM26 at 37 °C for 24 h. At predetermined time points (1, 2, 4, 8, 24 h), incubation media was aspirated, cells were washed with serum-free media, and membrane-bound and internalized radioactivity were collected using the method described earlier [43]. The membrane-bound radiolabelled conjugate were removed from cells by treatment with 4 M urea solution in a 0.1 M glycine buffer, pH 2, for 5 min on ice. The cell debris containing the internalized conjugate was detached by treatment with 1 M NaOH. Radioactivity of samples was measured, and percentage of membrane-bound, internalized and total radioactivity was calculated. The experiments were performed in triplicate.

Additionally, PC-3 cells were incubated with 1 nM of [^125^I]I-Tyr-PEG_2_-RM26 in presence of 20 mM sodium azide/10 mM 2-deoxyglucose at 37 °C for 8 h. Another set of dishes were incubated with 100 µM of chloroquine diphosphate and 20 nM of ammonium chloride in the presence of [^125^I]I-Tyr-PEG_2_-RM26 (1 nM). At predetermined time points (2, 4, and 8 h), media from a set of three dishes was removed, cells were detached by trypsin-EDTA solution, re-suspended, and radioactivity in cells was measured.

### 2.5. In Vivo Studies

Animal studies were planned and performed in agreement with EU Directive 2010/63/EU for animal experiments and Swedish national legislation concerning protection of laboratory animals. Experiments were approved by the Ethics Committee for Animal Research in Uppsala (C4/16, 26 February 2016). Biodistribution studies were performed in female BALB/C nu/nu mice purchased from Scanbur A/S (Karlslunde, Denmark). GRPR-expressing xenografts were established by subcutaneous injection of 5 × 10^6^ PC-3 cells/mouse in the right hind leg of mice 3 weeks before the experiment. The animals were randomized into groups of four mice for each data point. At the time of the experiment, the average animal weight was 16.3 ± 0.9 g. Average tumour weight was 0.09 ± 0.07 g.

#### 2.5.1. Biodistribution and In Vivo binding Specificity Test of [^125^I]I-Tyr-PEG_2_-RM26 and [^111^In]In-DOTA-PEG_2_-RM26 in BALB/c nu/nu Mice Bearing PC-3 Prostate Cancer Xenografts

To study tumour targeting, female BALB/c nu/nu mice bearing PC-3 xenografts (4 mice per data point) were intravenously co-injected into the tail vein with totally 40 pmol of [^125^I]I-Tyr-PEG_2_-RM26 and [^111^In]In-DOTA-PEG_2_-RM26 mixture (30 kBq, 100 µL in PBS). Mice were euthanized followed by dislocation of their neck at 0.5 h after injection and blood samples were collected by heart puncture. At 3 and 24 h after injection, mice were euthanized by an intraperitoneal injection of anaesthetic solution (20 µL of solution per gram of body weight: Ketalar, 10 mg/mL; Rompun, 1 mg/mL) followed by heart puncture. Salivary glands, thyroid, lung, liver, spleen, pancreas, stomach, small intestine, kidneys, tumour, muscle, and bone were collected and weighed. The organ radioactivity was measured in a gamma-counter and uptake values of organs were calculated as percentage of injected dose per gram tissue (% ID/g). To test the in vivo binding specificity, a group of mice was co-injected with 7.5 nmol of non-labelled peptide (NOTA-PEG_4_-RM26), and biodistribution was measured at 0.5 h after injection.

#### 2.5.2. Biodistribution of [^125^I]I-Tyr-PEG_2_-RM26 in NMRI Mice by Co-Injection of Phosphoramidon (PA) as In Vivo Stabilizer

Two groups of 4 mice were used for this experiment. Biodistribution of [^125^I]I-Tyr-PEG_2_-RM26 was evaluated in female NMRI mice (weight: 29.6 ± 1.7 g). Non-tumour bearing mice as a control group were injected with 40 pmol of [^125^I]I-Tyr-PEG_2_-RM26 (30 kBq, 100 μL in PBS) into the tail vein. Another group of mice was injected with same activity by co-injection of PA (15 µL of 20 mg/mL stock solution, 300 µg). After 0.5 h, mice were euthanized by dislocation of their neck, and blood samples were collected. Salivary glands, thyroid, lung, liver, spleen, pancreas, stomach, small intestine, kidneys, tumour, muscle, and bone were collected and weighed. The organ radioactivity was measured in a gamma-counter and uptake values of organs were calculated as percentage injected dose per gram tissue (% ID/g).

### 2.6. Statistical Analysis

Statistical treatment and linear regression analysis were performed using GraphPad Prism software version 5.00 for Windows, GraphPad Software, San Diego California. A two-tailed unpaired *t*-test was used for comparison of the two sets of data. The difference was considered as significant when *p* value was less than 0.05.

## 3. Results

### 3.1. Peptide Synthesis and Characterization

**Figure 2:**
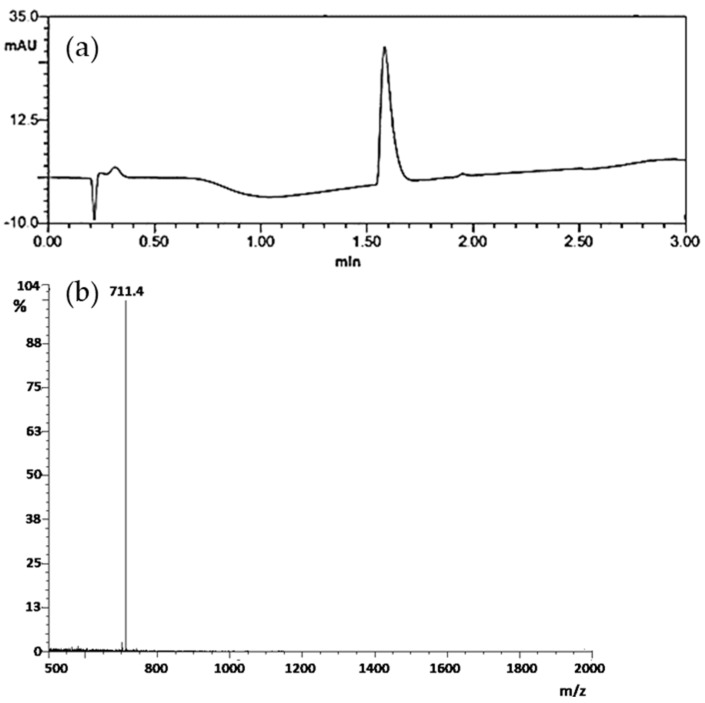
(**a**) High-performance liquid chromatography (HPLC) chromatogram of Tyr-PEG_2_-RM26 at 280 nm and (**b**) mass spectrum of Tyr-PEG_2_-RM26.

Tyr-PEG_2_-RM26 was synthesized as previously described and purified by preparative HPLC, generating in 1.7 mg of desired product, corresponding to a total yield of 6.0% (based on the initial loading of the resin). After LC-MS analysis, purity was determined, based on the 280 nm trace, to be over 95% (Figure 2a) and the m/z value found was in accordance with expected value ([M + 2H]^2+^ m/z = 711.4) (Figure 2b). The conjugate was freeze dried and used for in vitro and in vivo experiments. Figure 1 showed the structural formula of both Tyr-PEG_2_-RM26 and DOTA-PEG_2_-RM26.

### 3.2. Radiolabelling and In Vitro Stability Test

The radiolabelling of Tyr-PEG_2_-RM26 with ^125^I was performed at room temperature followed by HPLC analysis and preparative separation. The radiochemical yield was 77 ± 7% as determined using radio-HPLC. After purification using Sep-Pak C8 cartridge, the radiochemical purity was 95 ± 2%. The results of the in vitro stability test demonstrated that the radiolabelled conjugate was stable in PBS (95 ± 1%) and also in presence of excess amount of 1 mM NaI (92 ± 3%) after 1 h incubation at room temperature.

Labelling of DOTA-PEG_2_-RM26 with ^111^In resulted in radiochemical purity of 100%, and [^111^In]In-DOTA-PEG_2_-RM26 was used in biological experiments without additional purification.

### 3.3. Lipophilicity Assay: LogP

The lipophilicity of both radiolabelled conjugates was evaluated using “shake-flask”- method. Partition coefficient, log*P* of [^125^I]I-Tyr-PEG_2_-RM26 and [^111^In]In-DOTA-PEG_2_-RM26 was 1.0 ± 0.1 and −2.6 ± 0.4, respectively.

### 3.4. In Vitro Studies

#### 3.4.1. In Vitro Binding Specificity Assay

**Figure 3:**
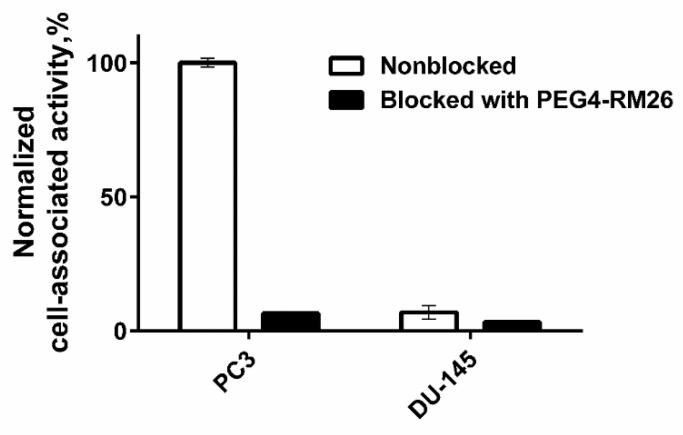
In vitro specificity of [^125^I]I-Tyr-PEG_2_-RM26 to PC-3 and DU-145 cells. Radiolabelled conjugate was added to cultured cells at a concentration of 1 nM. One set of culture dishes was pre-saturated with a 200-fold molar excess of non-labelled NOTA-PEG_4_-RM26 before incubation with the labelled conjugate.

In vitro binding specificity test demonstrated that binding of [^125^I]I-Tyr-PEG_2_-RM26 to PC-3 and DU-145 cells is receptor mediated. Pre-saturation of the cells with non-labelled NOTA-PEG_4_-RM26 significantly (*p* < 0.05) decreased the cell binding of the radiolabelled compounds (Figure 3). Specific binding to DU-145 cells was much lower than specific binding to PC-3 cells.

#### 3.4.2. In Vitro Competitive Binding Assay: IC_50_ Determination

**Figure 4:**
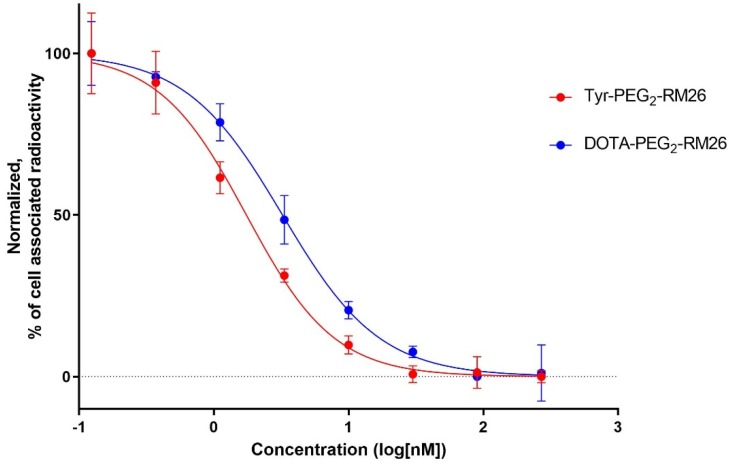
Inhibition of [^111^In]In-NOTA-PEG_4_-RM26 binding to PC-3 cells with X-PEG_2_-RM26 (X = tyrosine, DOTA). Data are presented as the mean value of three dishes ± SD.

Binding properties of Tyr-PEG_2_-RM26 and DOTA-PEG_2_-RM26 to PC-3 cells were compared in a competitive binding assay using [^111^In]In-NOTA-PEG_4_-RM26 as the displacement radioligand. The IC_50_ values for Tyr-PEG_2_-RM26 and DOTA-PEG_2_-RM26 were determined to be 1.7 ± 0.3 and 3.3 ± 0.5 nM, respectively (Figure 4).

#### 3.4.3. Cellular Processing Assay

**Figure 5:**
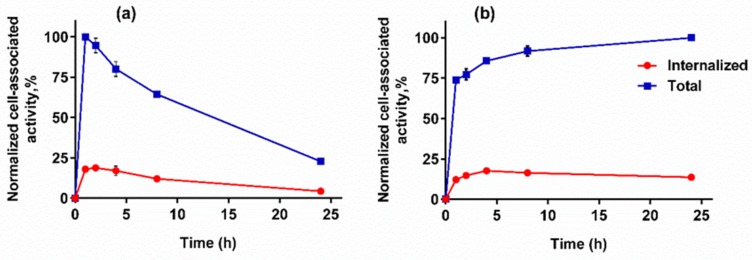
Cellular processing of (**a**) [^125^I]I-Tyr-PEG_2_-RM26 and (**b**) [^111^In]In-DOTA-PEG_2_-RM26 on PC-3. Data are presented as the mean values of three dishes ± SD. Not all error bars are visible due to the small standard deviations.

Data concerning cellular processing of [^125^I]I-Tyr-PEG_2_-RM26 and [^111^In]In-DOTA-PEG_2_-RM26 by PC-3 cells are presented in Figure 5. Cellular processing of [^125^I]I-Tyr-PEG_2_-RM26 by PC-3 cell line was characterized by fast binding and quite fast internalization during the first hour of incubation followed by a decreased cell-associated activity with time. The rapid binding of [^111^In]In-DOTA-PEG_2_-RM26 to PC-3 cells was accompanied by slower internalization, reaching 14% of cell-associated radioactivity after 24 h incubation at 37 °C.

**Figure 6:**
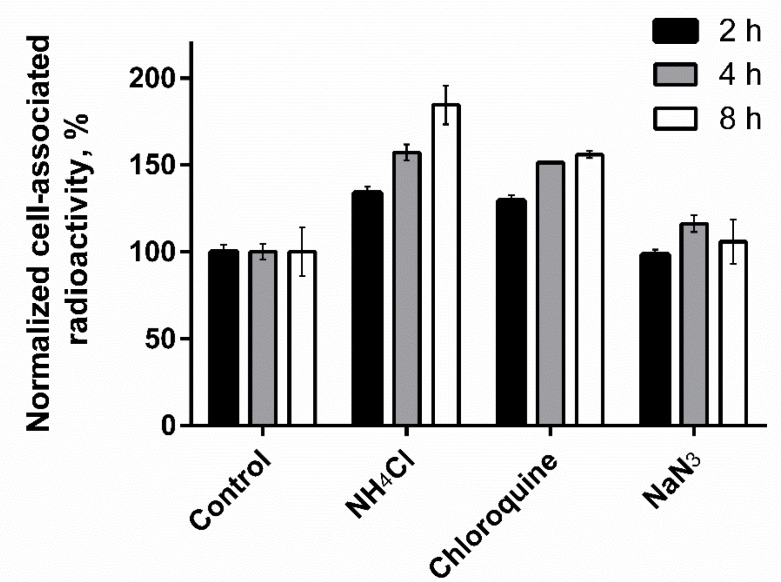
Normalized cell-associated radioactivity of [^125^I]I-Tyr-PEG_2_-RM26 in the presence 20 mM sodium azide/10 mM 2-deoxyglucose, 100 µM Chloroquine, 20 mM ammonium chloride by PC-3 cells during continuous incubation at 37 °C. One set of culture dishes was tested as control.

Figure 6 shows the effect of the lysosomotropic weak bases, chloroquine, and ammonium chloride, on amount of cell-bound activity of [^125^I]I-Tyr-PEG_2_-RM26 over time. Cell-bound activity and retention of [^125^I]I-Tyr-PEG_2_-RM26 on PC-3 cells in the presence of these two weak bases were considerably higher compared to control. Cell-associated activity for the cell treated with sodium azid, that should inhibit synthesis of the new receptors, was similar to the controls.

### 3.5. In Vivo Studies

#### 3.5.1. Biodistribution and In Vivo Binding Specificity Test of [^125^I]I-Tyr-PEG_2_-RM26 and [^111^In]In-DOTA-PEG_2_-RM26 in BALB/c nu/nu Mice Bearing PC-3 Prostate Cancer Xenografts

**Figure 7:**
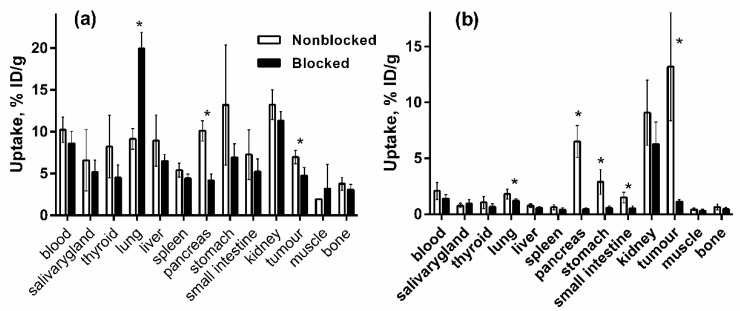
In vivo specificity of (a) [^125^I]I-Tyr-PEG_2_-RM26 and (b) [^111^In]In-DOTA-PEG_2_-RM26 in PC-3 tumour xenograft bearing BALB/C nu/nu at 30 min after injection. Blocking was performed by GRPR saturation with co-injection of non-labelled NOTA-PEG_4_-RM26. Asterisk (*) marks significant (*p* < 0.05) difference. Data are presented as a mean ± SD; *n* = 4. Error bars might not be seen because they are smaller than the symbols.

In vivo specificity of [^125^I]I-Tyr-PEG_2_-RM26 and [^111^In]In-DOTA-PEG_2_-RM26 binding to GRPR-expressing PC-3 in tumour xenografts was evaluated by saturation of receptors using a co-injection of non-labelled peptide (NOTA-PEG_4_-RM26) (Figure 7) at 0.5 h after injection. Uptake of [^125^I]I-Tyr-PEG_2_-RM26 in pancreas and tumour in the blocked group was significantly (*p* < 0.05) reduced compared to non-blocked group. For [^111^In]In-DOTA-PEG_2_-RM26, significant (*p* < 0.05) reduction of uptake for lung, pancreas, stomach, small intestine and tumour in the blocked group compared to non-blocked group was observed, indicating GRPR-specific accumulation of both radioconjugates. The blocking effect was more pronounced for [^111^In]In-DOTA-PEG_2_-RM26.

Biodistribution of [^125^I]I-Tyr-PEG_2_-RM26 and [^111^In]In-DOTA-PEG_2_-RM26 in PC-3-xenografted mice were studied in dual label study at 0.5, 3 and 24 h after injection and results are presented in Table 1. Data obtained for [^111^In]In-DOTA-PEG_2_-RM26 are in good agreement with previously published results [40]. Clearance of [^125^I]I-Tyr-PEG_2_-RM26 from blood was remarkably slower than clearance of [^111^In]In-DOTA-PEG_2_-RM26. The activity accumulation of [^125^I]I-Tyr-PEG_2_-RM26 in all normal organs was significantly (*p* < 0.05) higher than accumulation of [^111^In]In-DOTA-PEG_2_-RM26 at all three time points. High uptake was observed for the receptor-positive organs for both radioconjugates. Tumour uptake of [^111^In]In-DOTA-PEG_2_-RM26 was significantly (*p* < 0.05) higher than [^125^I]I-Tyr-PEG_2_-RM26, resulting in significantly higher tumour-to-organ ratio (Table 2). Moreover, the washout rate of [^111^In]In-DOTA-PEG_2_-RM26 from the target-expressing normal tissues was higher than that from the tumour, leading to increasing tumour-to-organ ratios over time.

#### 3.5.2. Biodistribution of [^125^I]I-Tyr-PEG_2_-RM26 in NMRI Mice by Co-Injection of Phosphoramidon (PA) as an In Vivo Stabilizer

**Figure 8:**
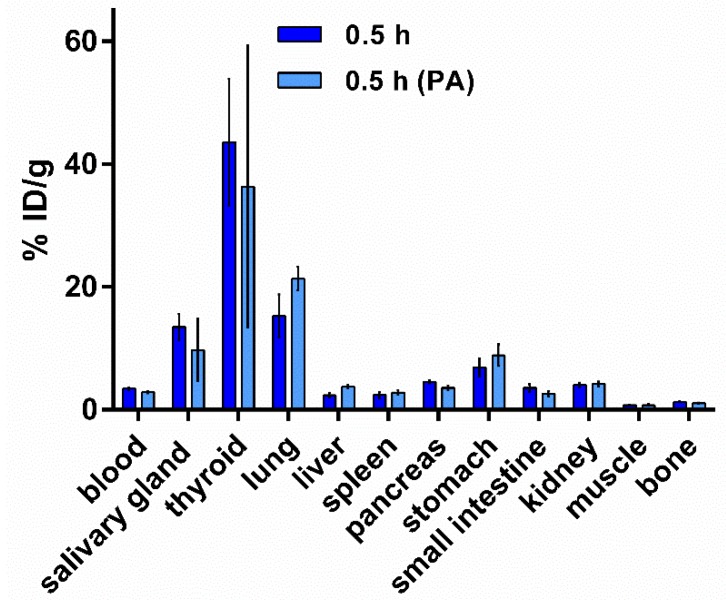
Effect of phosphoramidon (PA) on the biodistribution of [^125^I]I-Tyr-PEG_2_-RM26. Non-tumour bearing mice as the control group were intravenously injected with 40 pmol of [^125^I]I-Tyr-PEG2-RM26 (30 kBq, 100 μL in PBS) into the tail vein. Another group of mice was injected with same activity with co-injection of PA (15 µL of 20 mg/mL of stock solution, 300 µg).

Influence of co-injection of the enzyme inhibitor phosphoramidon (PA) on in vivo stabilization of the radiopeptide [^125^I]I-Tyr-PEG_2_-RM26 in non-tumour bearing mice was investigated. This strategy led to a remarkable enhanced uptake of the radiopeptides in tumour and receptor-expressing tissues, whereas uptake in most non-target organs and tissues was not affected. Results from the biodistribution study of [^125^I]I-Tyr-PEG_2_-RM26 are summarized in Figure 8. No significant increase of uptake in GRPR-positive organs (pancreas, small intestines, and stomach) was observed at 0.5 h after co-injection of PA. Moreover, co-injection of PA did not show any significant influence on blood clearance rate or uptake in normal non-targeted organs and tissues.

## 4. Discussion

The goal of this study was to test the hypothesis that use of non-residualizing radioiodine might offer advantages in radionuclide therapy when antagonistic, slowly internalizing bombesin analogues are used for targeting. For this purpose, bombesin analogue Tyr-PEG_2_-RM26 was synthesized, purified, and characterized (Figure 2). Tyr-PEG_2_-RM26 was successfully radiolabelled with ^125^I with good radiochemical yield and about 95% purity. The label was stable in PBS and under challenge with large molar excess of sodium iodide.

The lipophilicity test showed that [^125^I]I-Tyr-PEG_2_-RM26 is appreciably more lipophilic than [^111^In]In-DOTA-PEG_2_-RM26. All further biological tests in vitro and in vivo aiming at the comparison of [^125^I]I-Tyr-PEG_2_-RM26 and [^111^In]In-DOTA-PEG_2_-RM26 were performed with cells of the same passage and in a single batch of animals. Such design of the study eliminates uncertainties associated with possible changes of cell properties due to clonal selection during multiple passages and batch-to-batch variability of laboratory animals. [^125^I]I-Tyr-PEG_2_-RM26 maintained specific in vitro binding to GRPR-expressing cells, which was proportional to the expression level (2.5 × 10^5^ and 1.2 × 10^4^ receptors per cell for PC-3 and DU-145 [44], respectively (Figure 3). IC_50_-values for Tyr-PEG_2_-RM26 and DOTA-PEG_2_-RM26 were in the low nanomolar range (Figure 4). Binding of Tyr-PEG_2_-RM26 to GRPR (IC_50_ = 1.7 ± 0.3 nM) was somewhat stronger than binding of DOTA-PEG_2_-RM26 (IC_50_ = 3.2 ± 0.5 nM). This is in agreement with previous data showing that a negative charge on N-terminus is unfavourable for binding of bombesin analogues to GRPR [31,33].

The cellular processing of [^111^In]In-DOTA-PEG_2_-RM26 (Figure 5) was characteristic for radiometal-labelled antagonistic analogues of bombesin [32,33,34,35,36,37]. The internalization was slow reaching 14% of cell-associated radioactivity after 24 h incubation. Cell-associated activity of [^125^I]I-Tyr-PEG_2_-RM26 showed rapid increase during the first hour followed by decrease over time. Such type of cellular processing resembles the behaviour of radioiodinated GRPR-binding agonists, when rapid internalization is followed by intracellular degradation and leakage of radiometabolites. However, the internalization and efflux rates of [^125^I]I-Tyr-PEG_2_-RM26 were much slower than the rates for GRPR-binding agonists. Internalization of agonistic [^125^I]I-GRP and [^125^I]I-Tyr^4^-BBN was 75–80% within 20–40 min [19,45]. The decrease in cell-associated activity may be explained by lysosomal degradation of internalized radioiodinated conjugate followed by excretion of radiocatabolites and recycling of GRPR membrane surface [46,47]. These recycled GRPR can further bind and internalize [^125^I]I-Tyr-PEG_2_-RM26 existing in media. Although retention of cell-associated activity for [^125^I]I-Tyr-PEG_2_-RM26 was much worse than for the metal labelled RM26, it was better for this antagonistic agent than should be expected for agonistic ones. Efflux of 45–65% of cell associated activity was reported both for residualizing (^111^In-DOTA and ^99m^Tc((CO)_3_) and non-residualizing labels (^18^F-benzoate and ^125^I-Tyr) attached to agonists [19,48,49].

Previous studies have shown that use of lysosomotropic bases can reduce lysosomal degradation of radiohalogenated targeting agents and increase intercellular retention and uptake [50]. To check lysosomal degradation of [^125^I]I-Tyr-PEG_2_-RM26, treatment with lysosomotropic bases was performed. The results of this test showed significantly (*p* < 0.05) increased cell-bound activity and retention of [^125^I]I-Tyr-PEG_2_-RM26 in the presence of chloroquine or ammonium chloride (Figure 6). This confirms that internalization and lysosomal degradation play an essential role in the decrease of cell-associated activity of [^125^I]I-Tyr-PEG_2_-RM26. Overall, the results of the in vitro evaluation show very unusual cellular processing pattern for an antagonist. It is possible that coupling of a quite lipophilic tyrosine at N-terminus triggers internalization of [^125^I]I-Tyr-PEG_2_-RM26. It has to be noted that previous studies have demonstrated that introduction of the chelator may affect the functional (agonism/antagonism) profile of targeting peptides. For example, the antagonistic somatostatin analogue (H-Cpa-DCys-Asn-Phe-Phe-DTrp-Lys-Thr-PheThr-Cys-2Nal-NH_2_, 406-040-15) linked to DOTA as the chelator switched its pharmacological function to an agonist [51]. In that study, authors concluded that prediction of the influence of a peptide modification on its functional profile by adding a chelator is not possible and properties has to be tested experimentally. This study demonstrates for the first time that the internalization pattern of bombesin analogues might be changed by modification of the N-terminus. This may be an important information to consider in molecular design of radiolabelled peptides.

We have used a dual label approach for comparison of [^125^I]I-Tyr-PEG_2_-RM26 and [^111^In]In-DOTA-PEG_2_-RM26, when two labelled conjugates are co-injected in the same mice, and the uptake of different labels in tissue is determined by gamma-spectroscopy. In this way, all factors related to an animal (e.g., hormonal status, heart rate, renal function) and to a tumour (e.g., vascularization, vascular permeability, and a target expression level) influence biodistribution of both compounds in the same way. This permits to reveal a significant diffidence in biodistribution with relatively low number of animals. The in vivo specificity study (Figure 7) showed that uptake of both [^125^I]I-Tyr-PEG_2_-RM26 and [^111^In]In-DOTA-PEG_2_-RM26 in tumours and GRPR-expressing tissues was specific. By co-injection with an excess of non-labelled peptide, tumour uptake was significantly (*p* < 0.05) reduced from 13 ± 5% ID/g to 1 ± 0% ID/g for [^111^In]In-DOTA-PEG_2_-RM26 and from 7 ± 1% ID/g to 5 ± 1% ID/g for [^125^I]I-Tyr-PEG_2_-RM26, indicating specific GRPR-mediated uptake. There was a significant (*p* < 0.05) reduction of the uptake of both tracers in GRPR-expressing pancreatic tissue.

The biodistribution of [^125^I]I-Tyr-PEG_2_-RM26 and [^111^In]In-DOTA-PEG_2_-RM26 in mice was quite different. Biodistribution of [^111^In]In-DOTA-PEG_2_-RM26 was characterized by a rapid clearance of radioactivity from blood, GRPR-expressing organs, and other healthy organs and tissues, and the excretion of radioactivity was predominantly via kidney filtration. Clearance of activity from blood and other tissues after injection of [^125^I]I-Tyr-PEG_2_-RM26 was noticeably slower and there was an appreciable hepatic uptake. The high accumulation of activity in organs with expression of Na/I-symporters (thyroid, salivary glands and stomach) was observed for [^125^I]I-Tyr-PEG_2_-RM26, which indicates a re-distribution of radiometabolites. Tumor uptake for [^111^In]In-DOTA-PEG_2_-RM26 (13 ± 5% ID/g) was significantly (*p* < 0.05) higher than for [^125^I]I-Tyr-PEG_2_-RM26 (7 ± 1% ID/g) at 0.5 h after injection.

Tumor uptake of [^125^I]I-Tyr-PEG_2_-RM26 decreased more than 3-fold at 3 h compared to 0.5 h after injection, while decrease of tumour uptake was not so dramatic for [^111^In]In-DOTA-PEG_2_-RM26. The clearance of [^111^In]In-DOTA-PEG_2_-RM26 from non-targeted organs was very fast, which resulted in a significant (*p* < 0.05) higher tumour-to-organ ratio at 3 h after injection.

Due to the relatively small size of the peptides, modification of their structure can influence their affinity and selectivity for the targeted receptors. Previous studies of X-PEG_2_-RM26 (X = NOTA, NODAGA, DOTA, and DOTAGA) containing different chelators labelled with ^111^In and ^68^Ga showed that changing the radiometal and chelator can substantially influence the affinity and biodistribution profile [31,41]. All analogues showed antagonistic properties confirmed by the slow internalization rate after binding to the GRPR.

Degradation of radiopeptides, including bombesin-like peptides, by proteolytic enzymes present in blood, vasculature walls, liver, lungs, kidney, and gastrointestinal tract limits their successful application as theranostic probes [52,53]. These enzymes can potentially hamper radiopeptide-based imaging and therapy by cleaving radiopeptides into inactive radiometabolites. Previous studies have shown that co-injection of the enzyme inhibitor phosphoramidon (PA), can stabilize radiopeptides in vivo, resulting in a remarkable increase of tumour uptake, whereas uptake in non-target healthy organs and tissues is not affected [54]. The results of biodistribution of [^125^I]I-Tyr-PEG2-RM26 in non-tumourbearing mice demonstrated that co-injection of PA could not improve blood clearance rate or uptake in healthy organs and tissues. Thereby, degradation of radiolabelled conjugate cannot be the main reason for the high uptake in non-targeted organs and slow blood clearance rate.

Taken together, in vitro and in vivo data suggest that conjugation of tyrosine at N-terminus of PEG_2_-RM26 results in an appreciably more rapid internalization compared to the internalization of PEG_2_-RM26 with DOTA conjugated at N-terminus. The use of a non-residualizing radiohalogen label causes a poor retention of activity by malignant cells both in vitro and in vivo. Furthermore, blood clearance of [^125^I]I-Tyr-PEG_2_-RM26 is slower comparing to [^111^In]In-DOTA-PEG_2_-RM26. Studies with other peptides [55,56] suggest that slow clearance might be due to binding of blood proteins caused by an elevated lipophilicity. Overall, such features are undesirable for radionuclide therapy.

**Figure 9:**
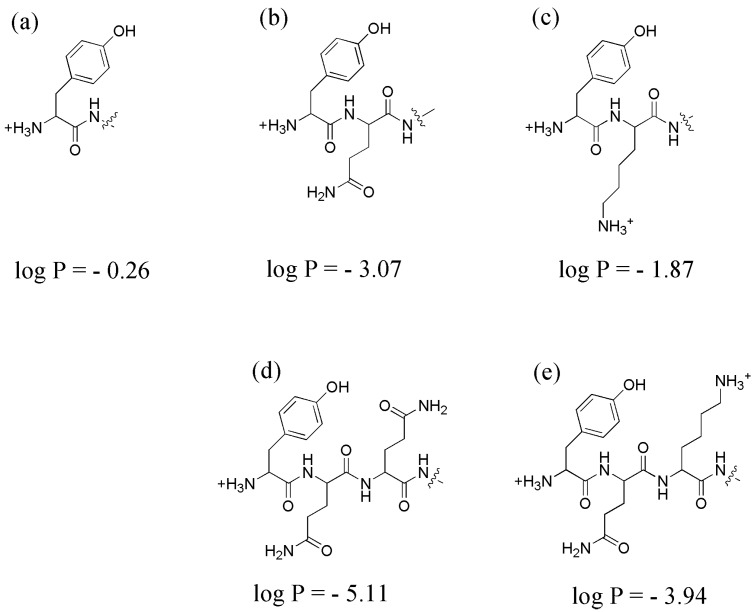
Structures of N-termini containing (**a**) Tyr; (**b**) Tyr-Gln; (**c**) Tyr-Lys; (**d**) Tyr-Gln-Gln; (**e**) Tyr-Gln-Lys and their log P calculated using ChemDraw Ultra.

The undesirable features of [^125^I]I-Tyr-PEG_2_-RM26 are highly likely associated with the presence of lipophilic side-chain of tyrosine at N-terminus. At the same time, the presence of phenolic moiety is critical for radioiodination and endowing of non-residualizing property of the label. Obviously, the improved molecular design of a GRPR antagonist with non-residualizing label should keep the presence of a tyrosine at N-terminus but foresee a compensation of its lipophilicity. Earlier studies have shown that increase of overall and local hydrophilicity of targeted polypeptides by incorporation of amino acids with polar or charged side-chain in a proximity to a label facilitates their blood clearance [57,58] and reduces hepatic uptake [57,58,59,60]. An incorporation of a glutamate had the strongest benign affect in these studies. However, both this and previous studies [33,34] show that a placement of negatively charged moiety decreases affinity of RM26 binding to GRPR. Therefore, we consider the use of amino acids with neutral polar or positively charged side-chains, i.e., glutamine and lysine. For preliminary assessment of hydrophilicity of the combination of these amino acids with tyrosine, logP of the oligopeptides was calculated using ChemDraw Ultra assuming amidated C-tremini (CambfidgeSoft, Cambridge, MA, USA) and compared with logP of tyrosine (Figure 9).

Apparently, addition of glutamine and lysine to tyrosine would enhance hydrophilicity of the N-terminus of tyrosine-containing RM26 analogue. Particularly a Tyr-Gln-Gln-combination looks promising. The obvious next step should be preparation of a small library of RM26 analogues containing both tyrosine and glutamine and their comparative evaluation.

## 5. Conclusions

The results showed that use of [^125^I]I-Tyr-PEG_2_-RM26 was not advantageously for radionuclide targeting of PC compared with [^111^In]In-DOTA-PEG_2_-RM26 as hypothesized. An introduction of a hydrophobic tyrosine group at N-terminus of RM26 changed cellular processing and biodistribution patterns of antagonistic bombesin analogue making them unfavourable for radionuclide therapy. Incorporation of amino acids with hydrophilic side-chains in the proximity to tyrosine might improve the biodistribution pattern of a radioiodinated tyrosine-containing RM26 analogue.

## Figures and Tables

**Table 1:** Biodistribution of [^125^I]I-Tyr-PEG_2_-RM26 and [^111^In]In-DOTA-PEG_2_-RM26 in GRPR-expressing PC-3 tumour xenografted female BALB/C nu/nu mice at three time points (total injected mass of 40 pmol). Data are presented as the mean percentage of injected dose per gram tissue (% ID/g; Mean ± SD; *n* = 4), NM stands for non-measureable.

	Uptake, % ID/g					
	0.5 h		3 h		24 h	
	[^125^I]I-Tyr-PEG_2_-RM26	[^111^In]In-DOTA-PEG_2_-RM26	[^125^I]I-Tyr-PEG_2_-RM26	[^111^In]In-DOTA-PEG_2_-RM26	[^125^I]I-Tyr-PEG_2_-RM26	[^111^In]In-DOTA-PEG_2_-RM26
blood	10.2 ± 1.5 ^a,d,e^	2.1 ± 0.8 ^a,g,h^	2.9 ± 1.5 ^b,d,f^	0.03 ± 0.00 ^b,g,i^	0.02 ± 0.00 ^c, e, f^	0.006 ± 0.002 ^c,h,i^
salivary gland	6.6 ± 3.7 ^a,d^	0.8 ± 0.2 ^a,g^	2.0 ± 1.2 ^b,d^	0.07 ± 0.02 ^b,g^	NM	NM
thyroid	8.2 ± 3.7 ^a,d^	1.1 ± 0.5 ^a,g^	2.2 ± 1.6 ^b,d^	0.1 ± 0.0 ^b,g^	NM	NM
lung	9.1 ± 1.2 ^a,d^	1.8 ± 0.4 ^a,g^	3.5 ± 1.4 ^b,d,f^	0.1 ± 0.0 ^b,g^	0.2 ± 0.1 ^f^	NM
liver	8.9 ± 3.1 ^a,d,e^	0.8 ± 0.2 ^a,g,h^	2.6 ± 1.1 ^b,d,f^	0.2 ± 0.0 ^b,g,i^	0.2 ± 0.0 ^c,e,f^	0.1 ± 0.0 ^c,h,i^
spleen	5.4 ± 0.8 ^a,d^	0.6 ± 0.2 ^a,g^	2.0 ± 0.8 ^b,d^	0.1 ± 0.0 ^b,g^	NM	NM
pancreas	10.1 ± 1.2 ^a,d^	6.5 ± 1.4 ^a,g^	2.3 ± 0.6 ^b,d^	0.2 ± 0.0 ^b,g^	NM	NM
stomach	13.2 ± 7.2 ^a,d^	2.9 ± 1.1 ^a,g^	4.6 ± 2.7 ^b,d,f^	0.4 ± 0.1 ^b,g,h^	0.1 ± 0.0 ^f^	NM
small intestine	7.3 ± 3.0 ^a,d^	1.5 ± 0.5 ^a,g^	1.8 ± 0.7 ^b,d^	0.2 ± 0.1 ^b,g^	NM	NM
kidney	13.2 ± 1.8 ^d,e^	9.1 ± 2.9 ^g,h^	6.1 ± 1.1 ^b,d^	2.4 ± 0.3 ^b,f,g,i^	1.5 ± 0.3 ^c,e,f^	0.8 ± 0.2 ^c,h,i^
tumour	7.0 ± 0.8 ^a,d^	13.2 ± 4.8 ^a,h^	2.1 ± 0.8 ^b,d^	9.6 ± 0.6 ^b,i^	NM	3.6 ± 1.3 ^h,,i^
muscle	2.0 ± 0.0^a,d^	0.4 ± 0.1 ^a,g^	0.6 ± 0.3 ^b,d^	0.02 ± 0.00 ^b,g^	NM	NM
bone	3.8 ± 0.8 ^a,d,h^	0.6 ± 0.3 ^a,g^	1.1 ± 0.5 ^b,d^	0.05 ± 0.02 ^b,g^	NM	NM

^a^ significant difference (*p* < 0.05) between [^125^I]I-Tyr-PEG_2_-RM26 and [^111^In]In-DOTA-PEG_2_-RM26 at 0.5 h after injection; ^b^ significant difference (*p* < 0.05) between [^125^I]I-Tyr-PEG_2_-RM26 and [^111^In]In-DOTA-PEG_2_-RM26 at 3 h after injection; ^c^ significant difference (*p* < 0.05) between [^125^I]I-Tyr-PEG_2_-RM26 and [^111^In]In-DOTA-PEG_2_-RM26 at 24 h after injection; ^d^ significant difference (*p* < 0.05) between 0.5 h ^125^I and 3 h ^125^I; ^e^ significant difference (*p* < 0.05) between 0.5 h ^125^I and 24 h ^125^I; ^f^ significant difference (*p* < 0.05) between 3 h ^125^I and 24 h ^125^I; ^g^ significant difference (*p* < 0.05) between 0.5 h ^111^In and 3 h ^111^In; ^h^ significant difference (*p* < 0.05) between 0.5 h ^111^In and 24 h ^111^In; ^i^ significant difference (*p* < 0.05) between 3 h ^111^In and 24 h ^111^In.

**Table 2:** Tumour-to-organ ratios of [^125^I]I-Tyr-PEG_2_-RM26 and [^111^In]In-DOTA-PEG_2_-RM26 in BALB/C nu/nu mice bearing GRPR-expressing PC-3 tumour xenograft at 0.5, 3 and 24 h after injection^;^ (Mean ± SD; *n* = 4), NM stands for non-measureable.

	Tumor-to-Organ Ratio					
	0.5 h		3 h		24 h	
	[^125^I]I-Tyr-PEG_2_-RM26	[^111^In]In-DOTA-PEG_2_-RM26	[^125^I]I-Tyr-PEG_2_-RM26	[^111^In]In-DOTA-PEG_2_-RM26	[^125^I]I-Tyr-PEG_2_-RM26	[^111^In]In-DOTA-PEG_2_-RM26
blood	0.7 ± 0.0 ^a,e^	6.3 ± 1.1 ^a,g,h^	0.8 ± 0.1 ^b,f^	373 ± 47 ^b,g,i^	6 ± 4 ^c,e,f^	672 ± 41 ^c,h,i^
salivary gland	1.3 ± 0.6 ^a^	16.5 ± 3.6 ^a,g^	1.2 ± 0.3 ^b^	146 ± 36 ^b,g^	NM	NM
thyroid	1.0 ± 0.5 ^a^	14.6 ± 5.8 ^a,g^	1.2 ± 0.6 ^b^	105 ± 40 ^b,g^	NM	NM
lung	0.8 ± 0.0 ^a,d^	7.1 ± 1.8 ^a,g^	0.6 ± 0.1 ^b,d^	123 ± 7 ^b,g^	1.0 ± 0.6	NM
liver	0.8 ± 0.2 ^a^	16.4 ± 3.8 ^a,g,h^	0.8 ± 0.2 ^b^	61 ± 3 ^b,g,i^	0.6 ± 0.5 ^c^	45 ± 8 ^c,h,i^
spleen	1.30 ± 0.2 ^a,d^	20.8 ± 2.5 ^a,g^	1.1 ± 0.1 ^b,d^	119 ± 7 ^b,g^	NM	NM
pancreas	0.7 ± 0.1 ^a^	2.0 ± 0.4 ^a,g^	0.9 ± 0.1 ^b^	50 ± 6 ^b,g^	NM	NM
stomach	0.6 ± 0.2 ^a^	4.6 ± 1.1 ^a,g^	0.5 ± 0.1 ^b^	27 ± 6 ^b,g^	NM	NM
small intestine	1.1 ± 0.6 ^a^	9.0 ± 3.3 ^a,g^	1.3 ± 0.4 ^b^	68 ± 35 ^b,g^	NM	NM
kidney	0.5 ± 0.0 ^a,d,e^	1.4 ± 0.3 ^a,g,h^	0.3 ± 0.1 ^b,d,f^	4 ± 0 ^b,g^	0.10 ± 0.0 ^c,e,f,i^	5 ± 1 ^c,h,i^
muscle	3.6 ± 0.4 ^a^	29.9 ± 6.6 ^a,g^	3.7 ± 0.6 ^b^	428 ± 41 ^b,g^	NM	NM
bone	1.9 ± 0.2 ^a^	22.8 ± 12.8 ^a,g^	1.9 ± 0.3 ^b^	221 ± 110 ^b,g^	NM	NM

^a^ significant difference (*p* < 0.05) between [^125^I]I-Tyr-PEG_2_-RM26 and [^111^In]In-DOTA-PEG_2_-RM26 at 0.5 h after injection; ^b^ significant difference (*p* < 0.05) between [^125^I]I-Tyr-PEG_2_-RM26 and [^111^In]In-DOTA-PEG_2_-RM26 at 3 h after injection; ^c^ significant difference (*p* < 0.05) between [^125^I]I-Tyr-PEG_2_-RM26 and [^111^In]In-DOTA-PEG_2_-RM26 at 24 h after injection; ^d^ significant difference (*p* < 0.05) between 0.5 h ^125^I and 3 h ^125^I; ^e^ significant difference (*p* < 0.05) between 0.5 h ^125^I and 24 h ^125^I; ^f^ significant difference (*p* < 0.05) between 3 h ^125^I and 24 h ^125^I; ^g^ significant difference (*p* < 0.05) between 0.5 h ^111^In and 3 h ^111^In; ^h^ significant difference (*p* < 0.05) between 0.5 h ^111^In and 24 h ^111^In; ^i^ significant difference (*p* < 0.05) between 3 h ^111^In and 24 h ^111^In.
